# Can Caring Create Prejudice? An Investigation of Positive and Negative Intergenerational Contact in Care Settings and the Generalisation of Blatant and Subtle Age Prejudice to Other Older People

**DOI:** 10.1002/casp.2294

**Published:** 2016-11-29

**Authors:** Lisbeth Drury, Dominic Abrams, Hannah J. Swift, Ruth A. Lamont, Katarina Gerocova

**Affiliations:** ^1^ Centre for the Study of Group ProcessesUniversity of KentUK; ^2^ School of PsychologyUniversity of ExeterUK; ^3^ PenCLAHRC, Institute of Health ResearchUniversity of Exeter Medical SchoolUK

**Keywords:** ageism, intergroup contact, dehumanisation, generalisation, negative contact, positive contact

## Abstract

Caring is a positive social act, but can it result in negative attitudes towards those cared for, and towards others from their wider social group? Based on intergroup contact theory, we tested whether care workers' (CWs) positive and negative contact with old‐age care home residents (CHRs) predicts prejudiced attitudes towards that group, and whether this generalises to other older people. Fifty‐six CWs were surveyed about their positive and negative contact with CHRs and their blatant and subtle attitudes (humanness attributions) towards CHRs and older adults. We tested indirect paths from contact with CHRs to attitudes towards older adults via attitudes towards CHRs. Results showed that neither positive nor negative contact generalised blatant ageism. However, the effect of negative, but not positive, contact on the denial of humanness to CHRs generalised to subtle ageism towards older adults. This evidence has practical implications for management of CWs' work experiences and theoretical implications, suggesting that negative contact with a subgroup generalises the attribution of humanness to superordinate groups. Because it is difficult to identify and challenge subtle prejudices such as dehumanisation, it may be especially important to reduce negative contact. © 2016 The Authors. *Journal of Community & Applied Social Psychology* Published by John Wiley & Sons Ltd.

Age‐related discrimination is reported more commonly than both gender and race‐related discrimination (Abrams, Eilola, & Swift, 2009). Despite legal protection against age discrimination in health and social care (Age Discrimination Act, 1975; Equality Act, 2010), many older people feel that they do not receive the treatment they deserve because of their age. Older people often report being treated with a lack of dignity and respect (Age UK, 2013). Further, government reviews of five areas of health and social care in the UK also support the conclusion that ageism continues within these settings (e.g. Centre for Policy on Ageing, 2009a, 2009b). Whilst problems of institutional discrimination can arise from structural factors (e.g. limited resources leading to age‐based rationing and difficulty with access to services), they can also occur ‘bottom up’, from the prejudices of individuals within an organisation (cf. Abrams, Swift, Lamont, & Drury, 2015; Swift, Abrams, Drury, & Lamont, 2016). Tackling institutional age discrimination therefore requires a better understanding of the experiences of those that work in these organisations and the contact they have with older people. In the present research, we focus on the institutional care of older people, an arena in which there are known problems of elder abuse and neglect.

Research reveals that care workers' (CWs) positive relationships with care home residents (CHRs) are associated with better CHR health (Leedahl, Chapin, & Little, 2015), whilst CWs' ageist attitudes are associated with psychological abuse of CHRs (Bonnie & Wallace, 2003; Weir, 2004). Prior research, however, has not explored the antecedents of CWs' attitudes to CHRs or older people more broadly, or how CWs' interaction experiences shape these attitudes. Using the social psychological theory of intergroup contact as a framework (Allport, 1954; Pettigrew & Tropp, 2006), the current study therefore explores how CWs' prior interactions with CHRs, both positive and negative, relate to their attitudes towards CHRs and whether these attitudes might generalise to older people more widely. Given the increasing number of older adults in social care, it is particularly important to understand how CWs' interactions with CHRs may predict their attitudes towards older adults in general.

### Intergroup contact and ageism

Allport's contact hypothesis (1954) holds that positive interactions between individuals from different groups reduce prejudice. Ideal conditions exist when interactions are intimate (e.g. friendships), when individuals have equal status, work towards common goals and have institutional support. Meta‐analytic evidence has established that positive contact reduces prejudice towards a range of social groups, including age groups (Pettigrew & Tropp, 2006).

Research exploring *intergenerational* contact demonstrates that young adults' ageist attitudes can be reduced by contact with older people in everyday encounters (Bousfield & Hutchison, 2010; Drury, Hutchison, & Abrams, 2016; Knox, Gekoski, & Johnson, 1986; Schwartz & Simmons, 2001). Workplace intergenerational contact has also been linked to reduced ageism (Allan & Johnson, 2009; for a review of intergenerational contact, see Drury, Abrams, & Swift, in press). However, there is a lack of evidence to suggest that these findings are generalisable to health and social care contexts where older adults are more likely to be dependent upon their younger counterparts.

Health and social care settings offer unique opportunities to study intergenerational contact in a context in which dependency varies. Moreover, it is possible for this contact to be both positive and negative and to either confirm or disconfirm stereotypes and ageist attitudes (Caspi, 1984). For instance, medical students running a health promotion programme at older adults' centres reported more negative attitudes towards older adults after the programme than before (Reinsch & Tobis, 1991). Yet, findings in health and social care settings are mixed, some research reports a reduction in negative attitudes (Meyer, Hassanein, & Bahr, 1980; Gomez, Otto, Blattstein, & Gomez, 1985), whilst other research finds no change (Eddy, 1986; Reyna, Goodwin, & Ferrari, 2007).

In sum, health and social care contact research does not consistently support the wider literature in which workplace contact reduces ageist attitudes (Allan & Johnson, 2009; Nochajski, Davis, Waldrop, Fabiano, & Goldberg, 2011; Van Dussen & Weaver, 2009). Additionally, little is known about the specific nature of CWs' ageist attitudes (Eymard & Douglas, 2012).

### Positive and negative intergroup contact

Most intergroup contact research focuses on positive contact (Pettigrew & Tropp, 2011), such as agreeable interactions with a member of a different social group. Conversely, negative contact is associated with threat to oneself or one's social group, can occur when contact is involuntary and is frequently reported by those who experience repeated contact, often in the workplace (Pettigrew & Tropp, 2011). For example, Dhont, Cornelis, and Van Hiel's (2010) study of police officers' workplace contact with illegal immigrants revealed that positive and negative contact were independently related to their prejudice towards immigrants as an outgroup.

A small body of research compares the effects of positive and negative contact. Analysis of a national probability sample of 1383 German citizens found that positive contact with foreigners (mainly Turkish Muslims) was more predictive of prejudice towards Muslims than negative contact (Pettigrew, 2008). Yet, alternative research suggests that negative contact may increase overt prejudice more than positive contact reduces it (Barlow et al., 2012). For example, Graf, Paolini, and Rubin (2014) examined Europeans' contact with individuals from neighbouring countries. Despite being a third as likely to occur, negative contact had a greater influence than positive contact on attitudes towards other national groups. These divergent findings mean that it is important to investigate both positive and negative contact when considering how contact may predict CWs' attitudes.

The present study measures CWs' positive and negative contact experiences with CHRs, and examines how these may predict attitudes towards CHRs. Extrapolating from prior research, we hypothesise that CWs will experience positive contact more than negative contact and both types of contact should predict attitudes towards CHRs.

### Generalisation of contact

The second question is whether attitudes towards CHRs, resulting from positive and negative contact, generalise to older people more widely. Pettigrew and Tropp's (2006) meta‐analysis of 515 intergroup contact studies supports the theory (Pettigrew, 1998) that positive contact generalises in three ways: (i) from an outgroup member to other outgroup members in different prejudice situations; (ii) from an individual outgroup member to the entire outgroup; and (iii) from a primary contact group to an independent secondary group. The current study will extend the second type of generalisation, by examining whether contact with an individual can be generalised to attitudes towards known outgroup members and in turn to the larger outgroup category.

As mentioned, Pettigrew and Tropp's (2006) meta‐analysis provides evidence that following positive contact, attitudes towards an immediate contact partner become more favourable and reliably extend to the contact partner's wider social group. Yet, alternative research suggests that negative contact may generalise more readily than positive contact because of a greater influence on social category salience (Paolini, Harwood, & Rubin, 2010). High category salience facilitates the generalisation of attitudes from an individual to the wider outgroup (Brown & Hewstone, 2005), and across two experiments Paolini et al. (2010) found that category salience was greater after negative compared to positive contact. Indeed, a field study found that negative contact with immigrant survivors of a natural disaster generalised to prejudice towards the wider immigrant outgroup via prejudice towards immigrant survivors (Vezzali, Andrighetto, Di Bernardo, Nadi, & Bergamini, in press).

Therefore, the literature presents mixed findings regarding the potential of positive and negative contact to extend to wider outgroups. As existing studies testing the generalisation of contact to outgroup attitudes have examined either only positive contact or negative contact individually (Pettigrew & Tropp, 2006;Vezzali et al., in press), we adopted a novel approach including both types of contact in the same study. We predicted that CWs' attitudes relating to their positive and negative contact with CHRs would generalise and shape their attitudes towards older adults, but that generalisation effects would occur more readily for negative, rather than positive contact.

### Intergenerational contact, blatant and subtle attitudes

Ageist attitudes can be looked at in a number of ways. Blatant attitudes are those of which respondents are aware and can articulate, whereas subtle attitudes refer to less obvious prejudice, such as expressing benevolent but patronising stereotypes that can be expressed without conscious desire to malign. Research using stereotype trait attribution measures shows that subtle ageism can be reduced by intergenerational contact (Schwartz & Simmons, 2001). Further analyses of national surveys revealed that across all ages, those with older friends are less likely to support the stereotype that incompetence increases with age (Tasiopoulou & Abrams, 2006). Yet, analysis of similar data revealed a weaker relationship between intergenerational friendships and blatant expressions of ageism (Vauclair, Abrams, & Bratt, 2010). These divergent findings suggest that respondents may be disinclined to express ageism blatantly, in which case subtle measures may be more sensitive and less susceptible to socially desirable responding. The present research therefore includes both blatant and subtle measures of ageism and sought to test whether both types of attitudes generalise from positive and negative intergenerational contact.

A type of subtle prejudice pertinent to older people is dehumanisation, which is defined as ‘the denial of full humanness to others’ (Haslam, 2006, p.252). Dehumanisation is commonly mentioned in gerontology literature; the eldercare setting is seen as dehumanising (Berdes, 1987), and healthcare professionals are accused of using dehumanising language with older people (Cayton, 2006). Yet, although qualitative research reports CHRs' experience of dehumanisation (Fiveash, 1998), there appears to be no quantitative evidence of dehumanisation of CHRs. Dehumanisation is reduced by intergroup contact (Capozza, Falvo, Di Bernardo, Vezzali, & Visintin, 2014), but to our knowledge no research has examined whether contact‐related dehumanisation generalises from one group to another.

### Hypotheses

In summary, the present research hypothesises that CWs will experience more positive than negative contact with CHRs and that both types of contact will predict blatant and subtle attitudes towards CHRs. Furthermore, it is predicted that CWs' attitudes relating to negative contact with CHRs will generalise to their attitudes towards older adults more readily than attitudes associated with positive contact.

## Method

### Participants

Questionnaires were distributed by hand and online to CWs at 22 residential eldercare homes across South East England. The homes provided care for older adults with varied levels of physical and psychological dependency. Participation was anonymous and confidential, and respondents were entered into a cash prize draw. Sixty‐two responses were received (response rate 7.2%), three were removed as they exceeded 60 years old (an age when an individual may be considered as becoming an older adult e.g. Abrams et al., 2009) and three were removed because of excessive missing data. A sample of 56 respondents remained (42 paper, 14 online) and were aged 19 to 60 years old (*M* = 40.41, *SD* = 12.25), including 50 women and six men. To ensure that we were measuring *inter*group attitudes and participants viewed older adults as an outgroup, we asked, ‘in your view, at what age do women [men] start being described as elderly?’ All responded with an age older than their own.

### Measures

#### Positive and negative contact

Positive and negative contact scales were adapted (Dhont et al., 2010; Heitmeyer, 2002), and further items added to reflect contact within the care context.

#### Positive contact with CHRs

Three items measured the quality of positive contact (*α* = .86) by asking how much contact could be described as ‘pleasant’, ‘friendly’ and ‘co‐operative’ (1 = *none*, 7 = *all*). The frequency of positive contact with CHRs (*α* = .88) was measured by asking, ‘during the contact you have with service users, how often do you’ in relation to five items; ‘have interesting conversations’, ‘share a joke’, ‘have positive experiences’, ‘learn something new from service users’ and ‘feel like you are sharing time with a good friend’ (1 = *never*, *7* = *very often*). The means of both scales were multiplied to create an overall index of positive contact with CHRs (possible range 1–49).

#### Negative contact with CHRs

Three items measured the quality of negative contact (*α* = .80) by asking how much CWs' contact with CHRs could be described as ‘unpleasant’, ‘unfriendly’ and ‘uncooperative’ (1 = *none*, 7 = *all*). The frequency of negative contact with CHRs (*α* = .79) was measured by asking, ‘during the contact you have with service users, how often do you have’ in relation to three items; ‘conflicts’, ‘negative experiences’ and ‘arguments’ (1 = *never*, *7* = *very often*). As with positive contact, the means of both scales were multiplied to create an overall index of negative contact with CHRs (possible range 1–49).

#### Ageism

Blatant and subtle ageism (measured as the denial of humanness) towards CHRs and older adults were measured.

#### Blatant ageism towards CHRs and older adults

Participants rated their feelings towards CHRs using six 7‐point scales with endpoints labelled with pairs of adjectives (e.g. cold‐warm) adapted from the General Evaluation Scale (Wright, Aron, McLaughlin‐Volpe, & Ropp, 1997). 1Because of administrative oversight, blatant ageism measures in the online questionnaire featured 9‐point scales. Responses to positively valenced items were recoded so that higher scores indicated more ageism. Averaged items formed a reliable index (*α* = .91). The CHR blatant ageism scale was repeated but in relation to non‐family older adults. Averaged items again formed an index (*α* = .89).

#### Subtle ageism towards CHRs and older adults

According to dehumanisation theory, outgroups may be denied humanness along two trait‐attribution dimensions (Haslam, 2006). Uniquely human traits distinguish humans from animals (e.g. broadminded) and human nature traits differentiate humans from inanimate objects (e.g. fun‐loving). Scales measuring the attribution of traits include both desirable and undesirable traits (Haslam, 2006). However, our measure included only desirable traits (see Haslam & Bain, 2007) as previous research examining young adults' attitudes found that they deny humanness to older coworkers by attributing low levels of desirable but not undesirable traits (Wiener, Gervais, Brnjic, & Nuss, 2014).

#### Denial of uniquely human traits

Using a 7‐point scale (1 = *not at all*, 7 = *very much*), participants indicated how much CHRs possessed the following characteristics: broadminded, conscientious, humble and polite. Reversed averaged items formed an index (*α* = .77). Higher scores indicated greater denial of uniquely human traits to CHRs.

#### Denial of human nature traits

Using a 7‐point scale (1 = *not at all*, 7 = *very much*), 2Because of administrative oversight, subtle ageism measures in the online questionnaire featured 5‐point scales. participants indicated how much CHRs possessed the following characteristics; active, curious, friendly, helpful and fun‐loving. Reversed averaged items formed a reliable index (*α* = .81). Higher scores indicated greater denial of human nature traits.

The denial of humanness scales were repeated using older adults as the target group. Again, reversed averaged items formed two indices of subtle ageism, denial of uniquely human traits to older adults (*α* = .79) and denial of human nature traits to older adults (*α* = .83), with higher scores indicating more ageism.

### Prior contact with older adults

Two items measured contact frequency (*α* = .76), with older adults outside of work on a 7‐point scale (1 = *none*, 7 = *a lot*) ‘In everyday life, how much contact do you have with elderly people?’ and ‘How many elderly people do you know?’ One item measured contact quality ‘When you meet elderly people do you think the contact is mainly…’ (1 = *negative*, 7 = *positive*). Frequency responses were averaged and multiplied by quality scores (possible range 1–49).

## Results

Attitude variables were standardised. To establish that the online subsample (*n* = 42) and paper subsample (*n* = 14) were equivalent, we compared means and variances on all measures that used the same response scales. These comparisons confirmed that there were no differences between the two samples 3
*T* tests compared paper and online populations (for *Ms* and *SDs* see Table 1) on the following variables; *participant* age *t* (54) = −0.38, *p* = .705; positive contact *t* (54) = −0.17, *p* = .862; negative contact *t* (53) = −0.03, *p* = .974; prior contact *t* (54) = −1.18, *p* = .240. A chi‐square test compared participant gender of the two populations *X* ^2^ = (1,56) = 0.25, *p* = .618. Therefore, the within‐sample standardised data were combined across the two subsamples.

### Amount and strength of contact experienced

In line with the first hypothesis, CWs experienced both positive and negative contact. Moreover, positive contact experienced with CHRs (*M* = 30.71, *SD* = 10.31) was significantly higher than negative contact (*M* = 7.27, *SD* = 6.39), *t* (54) = 11.73, *p* < .001, Cohen's *d* = 1.58. Consistent with intergroup contact theory, positive contact was negatively correlated with blatant ageism towards CHRs and older adults. Additionally, it was negatively related to the denial of uniquely human traits to CHRs and the denial of human nature traits to older adults. Negative contact was positively correlated with all types of ageism towards CHRs and the denial of uniquely human traits to older adults (Table 1).

**Table 1:** Means, standard deviations and correlations among contact, attitudinal and demographic variables

	1	2	3	4	5	6	7	8	9	10
1. Positive contact										
2. Negative contact	−.55***									
3. Blatant ageism CHRs	−.46***	.31*								
4.Uniquely human CHRs	−.27*	.36**	−.06							
5. Human nature CHRs	−.25†	.36**	.07	.70***						
6. Blatant ageism OAs	−.33*	.13	.64***	.05	.06					
7. Uniquely human OAs	−.26†	.34*	−.03	.59***	.34***	.004				
8. Human nature OAs	−.29*	.24†	.03	.47***	.56***	−.07	.62***			
9. Prior contact OAs	.28*	.05	−.28*	−.04	−.07	−.23	.04	−.04		
10. Participant age	−.03	.07	−.004	.16	.09	.06	.14	.13	.09	
11. Gender	−.09	.02	.002	.19	.17	.10	.10	−.02	.10	.36**
*M*	30.95	7.27	2.11_a_/2.29_b_	3.80_a_/3.46_b_	3.19_a_/3.73_b_	2.58_a_/2.38_b_	3.24_a_/3.66_b_	2.81_a_/3.90_b_	30.59	40.41
*SD*	10.38	6.39	1.48_a_/1.59_b_	1.11_a_/0.66_b_	1.03_a_/0.62_b_	1.60_a_/2.00_b_	1.14_a_/0.82_b_	0.87_a_/0.79_b_	13.10	12.25

*N* = 56. CHRs = care home residents. OAs = older adults. Correlations are significant to

† *p* < .10

* *p* < .05

** *p* < .01

*** *p* < .001. Gender: Male = 1, female = 2. a = *M* and *SD* for responses to paper questionnaire (*n* = 42) blatant and subtle ageism both measured on 7‐point scales. b = *M* and *SD* for online questionnaire (*n* = 14), blatant ageism recorded on 9‐point scales and subtle ageism recorded using 5‐point scales. *M* and *SD* for positive contact vary from the statistics reported in the *t* test, because of one case deleted listwise as one participant did not complete the negative contact measure.

Multiple regression analyses tested relationships between contact with CHRs and ageism. All variables were standardised and in addition to positive and negative contact with CHRs, participant age, gender and prior contact with older adults were entered into the model as predictors. Because of the low sample size, we used bootstrapped analysis with 5000 bootstraps. Consistent with intergroup contact theory, positive contact marginally predicted lower blatant ageism towards CHRs *B* = −.31 (*SE* = .18), *p* = .092, 95% CIs [−.68, .03] and lower denial of human nature traits to older adults *B* = −.26 (*SE* = .15), *p* = .082, 95% CIs [−.53, .04], but did not predict other ageism towards CHRs or older adults (see Table 2 for all regressions). Negative contact significantly predicted higher denial of uniquely human traits to CHRs *B* = .30 (*SE* = .14), *p* = .039, 95% CIs [.01, .57], denial of human nature traits to CHRs *B* = .33, (*SE* = .16), *p* = .033, 95% CIs [.02, .63] and denial of uniquely human traits to older adults *B* = .28, (*SE* = .14), *p* = .033, 95% CIs [.01, .55] but did not predict other ageism towards CHRs or older adults. In summary, in contrast to prior research (Barlow et al., 2012) positive contact was more, rather than less, strongly related to blatant ageism towards CHRs than was negative contact. However, negative contact was more strongly and consistently related to subtle ageism.

**Table 2:** Bootstrapped regressions of positive and negative contact and covariates on attitudes towards CHRs and older adults

	Blatant ageism				Denial of uniquely human traits				Denial of human nature traits			
	B	*SE*	*p*	*95*% *CIs*	B	*SE*	*p*	*95*% *CIs*	B	*SE*	*p*	*95*% *CIs*
Attitudes towards CHRS												
Positive contact	−.31	.18	.092	−.68, .03	−.10	.24	.699	−.54, .36	−.05	.23	.821	−.50, .41
Negative contact	.15	.19	.431	−.22, .54	.30	.14	.039	.01, .57	.33	.16	.033	.02, .63
Age	.01	.13	.922	−.27, .25	.09	.14	.567	−.19, .37	.01	.16	.939	−.31, .32
Gender	−.02	.13	.900	−.27, .25	.14	.11	.199	−.06, .40	.15	.09	.102	−.02, .35
Prior contact	−.20	.14	.181	−.48, .08	−.05	.16	.776	−.36, .26	−.09	.17	.626	−.43, .24
Attitudes towards older adults												
Positive contact	−.27	.19	.166	−.71, .07	−.09	.15	.535	−.37, .22	−.26	.15	.082	−.53, .04
Negative contact	−.01	.16	.919	−.34, .29	.28	.14	.033	.01, .55	.09	.13	.476	−.17, .33
Age	.04	.14	.798	−.27, .30	.10	.13	.449	−.17, .36	.15	.15	.321	−.14, .42
Gender	.08	.10	.415	−.12, .29	.05	.17	.651	−.19, .32	−.10	.14	.437	−.37, .17
Prior contact	−.16	.17	.355	−.48, .19	.04	.13	.769	−.23, .29	.03	.15	.852	−.27, .31

*CI* = Bootstrapped confidence intervals. Gender; male = 1, female = 2. CHRs = care home residents.

### Generalisation effects via attitudes towards CHRs

To test the generalisation hypotheses, we used PROCESS model 4 (Hayes, 2013) with 5000 bootstraps to test indirect effects of contact with CHRs on ageism towards older adults via ageism towards CHRs. Again, we controlled for participant age, prior contact, gender and the other contact valence. We chose this analytical method as we specifically wanted to examine attitudes that generalised from contact with CHRs to attitudes towards older adults, *via* their effect on attitudes towards CHRs.

### Blatant ageism

We tested the indirect effects of positive and negative contact on blatant ageism towards older adults via blatant ageism towards CHRs. There was neither a significant indirect effect of positive contact −.18 (*SE* = .12), 95% CIs [−.49, .01], nor of negative contact .07 (*SE* = .12), 95% CIs [−.15, .32]. (See Table 3 for all indirect model coefficients.)

**Table 3:** Bootstrapped regressions from indirect effects models

	Blatant ageism					Denial of uniquely human traits					Denial of human nature traits				
	*CE*	*SE*	*t*	*p*	*CIs*	*CE*	*SE*	*t*	*p*	*CIs*	*CE*	*SE*	*t*	*p*	*CIs*
Total effects model															
Positive contact	−.22	.17	−1.33	.189	−.56, .11	−.09	.17	−0.53	.596	−.44, .25	−.26	.18	−1.46	.150	−.61, .10
Negative contact	.04	.16	0.27	.786	−.28, .37	.28	.16	1.73	.090	−.05, .61	.09	.17	0.52	.605	−.25, .42
Age	.11	.14	0.80	.429	−.17, .40	.10	.14	0.70	.484	−.18, .38	.15	.14	1.04	.302	−.14, .44
Gender	.05	.14	0.35	.728	−.23, .33	.05	.14	0.38	.706	−.23, .34	−.10	.15	−0.69	.494	−.39, .19
Prior contact	−.24	.14	−.1.68	.099	−.52, .05	.04	.14	0.27	.785	−.25, .32	.03	.14	0.18	.855	−.26, .32
Direct effects model															
Attitudes towards CHRs	.58	.13	4.59	.000	.32, .83	.55	.12	4.44	.0001	.30, .80	.57	.13	4.52	.000	.32, .82
Positive contact	−.04	.14	−0.30	.764	−.33, .25	−.04	.15	−0.26	.796	−.33, .26	−.23	.15	−1.52	.135	−.53, .07
Negative contact	−.03	.14	−0.18	.855	−.30, .25	.12	.14	0.83	.411	−.17, .41	−.10	.15	−0.69	.495	−.40, .20
Age	.08	.12	0.69	.491	−.16, .32	.05	.12	0.44	.666	−.19, .29	.14	.12	1.18	.246	−.10, .39
Gender	.07	.12	0.61	.547	−.16, .30	−.02	.12	−0.18	.858	−.27, .22	−.19	.12	−1.51	.138	−.44, .06
Prior contact	−.13	.12	−1.09	.281	−.37, .11	.06	.12	0.53	.599	−.18, .31	. 08	.12	0.61	.544	−.17, .32

*CE* = coefficient. *CI* = 95% confidence intervals. Total effect model includes covariates: age, gender, prior contact and opposite contact valence. Direct effects model repeats total effect model plus attitudes towards CHRs as mediator variable. IV = independent variable. DV = dependent variable.

### Subtle ageism

Next, we tested the hypotheses that contact with CHRs would generalise the denial of uniquely human and human nature traits to older adults.

#### Denial of uniquely human traits

**Figure 1:**
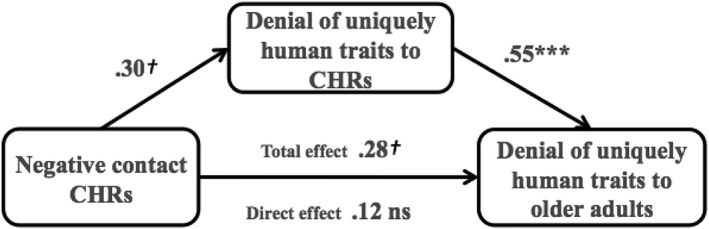
Indirect effect of negative contact on the denial of uniquely human traits to older adults. *Note*: Non standardized regression coefficients. ns = non‐significant, †*p* < .10, ***p < .001.

The indirect effect of positive contact on the denial of uniquely human traits to older adults through the denial of uniquely human traits to CHRs was non‐significant −.05 (*SE* = .16), 95% CIs [−.41, .21]. There was, however, a significant indirect effect of negative contact on the denial of uniquely human traits to older adults through of the denial of uniquely human traits to CHRs .16 (*SE* = .08), 95% CIs [.01, .35] (Figure 1) in which the total effect of negative contact on the denial of uniquely human traits to older adults was fully explained by the effect of negative contact on the denial of uniquely human traits to CHRs. More negative contact was associated with more subtle ageism towards CHRs, which in turn predicted subtle ageism towards older adults.

#### Denial of human nature traits

**Figure 2:**
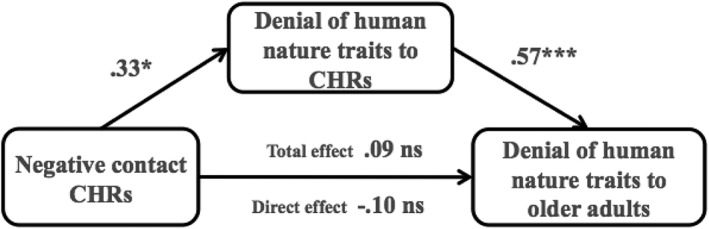
Indirect effect of negative contact on the denial of human nature traits to older adults. *Note*: Non standardized regression coefficients. ns = non‐significant, *p < .05, ***p < .001.

The indirect effect of positive contact on the denial of human nature traits to older adults through the denial of human nature traits to CHRs was non‐significant −.03 (*SE* = .15), 95% CIs [−.34, .24]. There was, however, a significant indirect effect of negative contact on the denial of human nature traits to older adults through the denial of human nature traits to CHRs .19 (*SE* = .11), 95% CIs [.001, .42] (Figure 2). The pattern was the same as that found for the denial of uniquely human traits.

In summary, the results provide evidence that negative contact plays a significant role in the generalisation of both types of subtle ageism.

## Discussion

The current research presents a novel test of whether positive and negative contact with an outgroup member independently predict not only attitudes towards that group, but attitudes towards others considered to be part of the wider outgroup. This was tested in the context of intergenerational contact between CWs and CHRs in an eldercare setting. It was predicted that contact between CWs and CHRs would be experienced by CWs more often as positive than negative contact, that each type of contact would predict CWs' blatant and subtle prejudice to CHRs, but that those attitudes would generalise to older adults as an outgroup more readily as a result of negative contact than positive contact.

As predicted, CWs experienced more positive than negative contact with CHRs. When controlling for the effects of prior contact, age, gender and the opposite contact valence, greater positive contact marginally predicted reduced blatant ageism towards CHRs, whilst greater negative contact significantly predicted the denial of uniquely human traits and human nature traits to CHRs. Therefore, the denial of humanness to CHRs was predicted by negative (but not positive) contact experiences. Positive contact directly generalised to predict a marginal decrease in the denial of human nature traits to older adults, and negative contact directly generalised to predict a significant increase in the denial of uniquely human traits to older adults. In the final analysis, only negative (not positive) contact indirectly predicted ageism towards older adults via attitudes towards CHRs, and this was evident on just subtle, and not blatant measures of ageism.

### The effects of positive contact and negative contact on ageism

This study increases understanding of positive and negative contact experienced in a previously unexplored context, residential social care for older adults. In line with prior research, we observed that individuals experience both types of contact in their workplace (cf. Dhont et al., 2010). Moreover, consistent with the caring role, positive contact was experienced more than negative contact (cf. Graf et al., 2014).

The present research is the first to show that negative contact predicts the denial of humanness to contact partners and illuminates how blatant and subtle forms of prejudice might be differently associated with contact. However, the denial of humanness reported was less than apparent for other outgroups (see Loughnan & Haslam, 2007). This may be partly because of CWs' internal values and positive motivation to interact with CHRs. Despite this, the denial of humanness reported in this study reflected more consistent and stronger levels of ageism than outcomes on blatant measures. A reason for the weaker effect of contact on blatant attitudes may be that it is a less sensitive measure of ageism in the social care context. Given the sensitive nature of older adult care, CWs' blatant ageism may partly reflect socially desirable responding.

Findings also provide understanding of how positive and negative contact are associated with blatant and subtle prejudice. Only positive contact predicted lower blatant ageism towards CHRs (marginally), whilst negative contact significantly predicted subtle ageism via the denial of both uniquely human and human nature traits to CHRs. A lack of equal status between CWs and CHRs may explain the weaker effects of positive contact in this context. This notion supports Allport's (1954) condition that equal group status is required for positive contact to successfully reduce prejudice. The findings suggest that even if experiences of positive contact may be associated with more favourable attitudes, experiences of negative contact are associated with higher levels of subtle prejudice. More concretely, following positive contact, CWs may feel friendly or warm towards CHRs, but following negative contact CWs attribute less humanness to CHRs. This different pattern of associations between prejudice and positive and negative contact could illuminate the divergent findings in previous research comparing the effects of positive to negative contact (Barlow et al., 2012; Graf et al., 2014; Pettigrew, 2008). It suggests that positive and negative contact may have more nuanced variations in their relationships with prejudice, depending upon the way in which prejudice is measured and manifested.

Prior research has mainly addressed racial or ethnic rather than age prejudice, so it is also possible that some aspects of age prejudice and age contact are distinct from those affecting other intergroup relationships (cf. Drury et al., 2016). Contact with CHRs seems less likely to involve aspects of intergroup threat and anxiety that can arise from interracial contact. Furthermore, research suggests that groups evaluated as warm but incompetent, such as older adults, are more likely to be passively harmed, whilst groups perceived as competent or competitive are more likely to be actively harmed (Cuddy, Fiske, & Glick, 2007). Both forms of harm can be serious. The potential for passive harm may not be reflected in blatant measures of prejudice such as the General Evaluation Scale (Wright et al., 1997), but may be signalled more clearly by subtle measures, as reflected in the relationship between negative contact and the denial of humanness.

### Generalisation of negative and positive contact

Previous research demonstrates that positive and negative contact extends from contact partner to general outgroup (Pettigrew, 2008; Vezzali et al., in press) and that because of its effect on group membership salience, negative contact may have stronger potential to generalise attitudes (Paolini et al., 2010). To our knowledge, the present research is the first to test the potential generalisation of attitudes from both positive and negative contact within the same study. Therefore, our analysis presents a clearer picture of the unique portion of variance in attitudes explained by contact of either positive or negative valence. The results do not replicate the generalisation of positive contact (Pettigrew, 2008), but do provide support for the generalisation of negative contact (Vezzali et al., in press). Therefore, the current findings are consistent with Paolini et al.'s (2010) conclusion that negative contact is more enduring than positive contact.

The present research additionally supports the contention that contact with a subgroup can generalise to predict prejudice to a superordinate group via prejudice towards the subgroup. Arguably, CHRs constitute a subgroup of an older adult superordinate group. In professional–public contact situations such as those between CWs and CHRs, or between the police and the public, it would be reasonable for professionals to assume the contact partner is a special case. Professionals could perceive these individuals either as sub‐types of the larger category (e.g. older people with dementia, illegal immigrants) or as completely separate categories (people with dementia versus people without dementia, criminals versus the general public). In further support of this hypothesis, Vezzali et al. (in press) demonstrated an indirect effect of negative contact with immigrant survivors of a natural disaster to the wider group of immigrants (superordinate group) *via* attitudes towards the immigrant survivors (subgroup). Future research should test if contact with individuals explicitly categorised as belonging to a subgroup predicts attitudes towards the superordinate group *via* attitudes towards the subgroup.

### Generalisation of blatant and subtle prejudice

Although we found no significant evidence for generalisation from positive and negative contact to *blatant* prejudice, the present evidence yielded the novel findings that negative contact may generalise in terms of *subtle* prejudice in the form of the denial of humanness. This finding is consistent with the idea that generalisation of contact effects may depend upon the type of prejudice under consideration, specifically that negative contact, albeit less frequent, may have a deeper impact on certain forms of prejudice (Barlow et al., 2012; Graf et al., 2014). Furthermore, it suggests that an additional reason for the durability of negative contact (Paolini et al. 2010) could be its impact on subtle forms of prejudice.

### Applied implications

The generalisation of negative attitudes arising from negative contact with older people is particularly important in the social care context. CWs are a group of adults who have higher than average levels of contact with older people and may well be a conduit through which other young adults learn about older adults (CWs provide indirect or ‘extended’ contact experiences for other young adults). Moreover, when CWs have negative contact, there is clearly a risk that it will generalise to elevate their subtle prejudice towards other older adults in general.

Although we did not find support for the hypothesis that positive contact experiences would be associated with lower prejudice, we are aware that the small sample size may have been insufficient to detect such relationships. More research is needed to improve confidence in conclusions about the effects of positive contact in this context. At present, however, the evidence only permits the conclusion that the benefits of positive contact with CHRs may not spread to older people in general, whereas the disadvantages of negative contact appear to do so.

This is the first empirical research to measure CWs' denial of humanness to CHRs and older adults, and provides some explanation of why CWs may dehumanise to different degrees. The findings reinforce qualitative evidence that CHRs may be vulnerable to dehumanising behaviour (Berdes, 1987; Cayton, 2006; Fiveash, 1998). It has been suggested that dehumanisation facilitates medical decisions and reduces staff stress (Lammers & Stapel, 2011) and is therefore functional (Vaes & Muratore, 2013), but the present study suggests, for the first time, some wider damaging effects of dehumanisation. Our findings show that when negative contact stimulates the denial of humanness to CHRs, this subtle negative attitude can also generalise to other older adults. This suggests that although dehumanisation may aid medical staff by facilitating disengagement when making difficult decisions relating to end of life care of older patients, it may permeate to affect their attitudes towards older adults in the wider community that are in good health. Perhaps, the relationship between contact and dehumanisation observed by Vaes and Muratore (2013) did not reflect a functional reaction but rather that much of the contact is negative. Thus, rather than accepting dehumanisation as an inevitable functional reaction to contact in health and social care contexts, efforts could be made to reduce the negative aspects of the contact and other sources of stress arising from the contact.

The findings also suggest that CWs' ageism towards CHRs (Bonnie & Wallace, 2003) is related to negative relationships experienced between CWs and CHRs within care homes. Future research should investigate the specific features of contact that make the experience negative in these contexts. This would facilitate the design of interventions to reduce ageism by targeting the particular types of encounter that are most likely to be negative within CWs' daily work schedules. This research also offers insight into how far reaching the effects of care work could be for ageism in society. It is important that detrimental effects produced by negative relationships within health and social care settings are fully understood and addressed in order to attenuate their effects on attitudes towards older adults more generally.

### Limitations and future research

Like the majority of studies of intergroup contact, the ability to make strong causal inferences from the present data is restricted because the data are correlational. However, the research is grounded in well‐developed theory that is supported (in other domains of contact) by plenty of experimental and longitudinal evidence (Pettigrew & Tropp, 2011). Because of the low pay and long working hours of CWs, and perhaps the sensitive nature of the research topic, acquiring access to large samples is challenging in this area. Despite these limitations, confidence in the meaningfulness of the present evidence is bolstered by the fact that the measures are internally reliable, and that relationships among variables are consistent with those observed in the wider intergroup contact literature. However, we recognise that the relationships among variables revealed in this study merit further investigation with larger samples, longitudinal designs and across varied health and social care contexts. Future research should also explore boundary conditions for the generalisation of contact effects on ageism towards older people more widely. For example, the degree to which CWs perceive a status imbalance between themselves and CHRs should be examined. The effects of positive contact are attenuated by unequal group status (Allport, 1954; González & Brown, 2003; Pettigrew & Tropp, 2006), but research is yet to examine how group status interacts with the generalisation of negative contact.

A limitation of this study was that it measured the denial of desirable, but not undesirable, human traits (Haslam & Bain, 2007). Research measuring both trait types would facilitate consistency and comparison with the wider dehumanisation literature (for a review see Haslam & Loughnan, 2014). For the wider literature on intergroup contact, it would be useful to test other instances of generalisation of subgroup contact to superordinate attitudes with other outgroups, for example in the case of ethnicity, sexuality and other stigmatised groups. In particular, it would be interesting to know whether generalisation depends on particular links or similarities between groups (e.g. across dependent or paternalised groups but not between dependent and competitive/non‐paternalised groups [cf. Abrams, Houston, Van de Vyver, & Vasiljevic, 2015]) and whether generalisation is moderated by the amount of threat or anxiety aroused by different groups. This is particularly important when the subgroup confirms negative aspects of the superordinate stereotype to a greater degree than the wider outgroup (e.g. immigrant prisoners versus immigrants in general). Additionally, the degree to which the subgroups are sub‐typed or treated as a distinct category from the superordinate group may affect outcomes. Arguably, generalisation may occur more readily if the contact partner is sub‐typed than if treated as a completely independent category.

### Summary

For the first time, this research provides evidence for the generalisation of the denial of humanness stemming from negative contact. Generalisation was not apparent in relation to positive contact and when measuring blatant attitudes as the outcome, showing not only the independent and distinct nature of negative and positive contact, but also the disparity between explicit and more subtle expressions of prejudice. Beyond these theoretical contributions, CWs' attitudes may affect institutional ageism because of the widespread use of health and social care and a lack of meaningful intergenerational contact. More widely, this research highlights the potential for negative contact in occupational settings to generalise to wider outgroups in the form of subtle dehumanising attitudes.
